# The role of anterior and posterior insula in male genital response and in visual attention: an exploratory multimodal fMRI study

**DOI:** 10.1038/s41598-020-74681-x

**Published:** 2020-10-28

**Authors:** Nicoletta Cera, João Castelhano, Cátia Oliveira, Joana Carvalho, Ana Luísa Quinta Gomes, Maria Manuela Peixoto, Raquel Pereira, Erick Janssen, Miguel Castelo-Branco, Pedro Nobre

**Affiliations:** 1 CPUP, Faculty of Psychology and Educational Sciences, University of Porto, Porto, Portugal; 2 CIBIT, Coimbra Institute for Biomedical Imaging and Translational Research, University of Coimbra, Coimbra, Portugal; 3 ICNAS, Faculty of Medicine, University of Coimbra, Coimbra, Portugal; 4 Digital Human-Environment Interaction Lab, Lusofona University, Porto, Portugal; 5 Centro de Investigação em Psicologia para o Desenvolvimento Positivo, Universidade Lusíada, Porto, Portugal; 6 Institute for Family and Sexuality Studies, Department of Neurosciences, University of Leuven, Leuven, Belgium

**Keywords:** Emotion, Sexual behaviour

## Abstract

Several studies highlighted the role of insula on several functions and in sexual behavior. This exploratory study examines the relationships among genital responses, brain responses, and eye movements, to disentangle the role played by the anterior and posterior insula during different stages of male sexual response and during visual attention to sexual stimuli. In 19 healthy men, fMRI, eye movement, and penile tumescence data were collected during a visual sexual stimulation task. After a whole-brain analysis comparing neutral and sexual clips and confirming a role for the bilateral insulae, we selected two bilateral seed regions in anterior and posterior insula for functional connectivity analysis. Single-ROI-GLMs were run for the FC target regions. Single-ROI-GLMs were performed based on areas to which participants fixate: “Faces”, “Genitals,” and “Background” with the contrast “Genitals > Faces”. Single-ROI-GLMs with baseline, onset, and sustained PT response for the sexual clips were performed. We found stronger effects for the posterior than the anterior insula. In the target regions of the posterior insula, we found three different pathways: the first involved in visual attention, onset of erection, and sustained erection; the second involved only in the onset of erection, and the third limited to sustained erection.

## Introduction

Sexual arousal (SA) is a multidimensional experience, comprising both peripheral and central processes^1–6^. Previous studies highlighted a complex set of regions involved in the processing of sexual stimuli and genital response. Cortical regions like the anterior cingulate cortex (ACC), insulae, dorsolateral prefrontal cortex (dlPFC), parietal cortices and subcortical regions, such as nucleus accumbens, putamen, hypothalamus, and thalamus play different roles, at different stages of male sexual arousal^7,8^. Georgiadis and Kringelbach^9^ proposed that sexual response in men involves a three-stage -cycle that depends on: wanting, liking, and inhibition^10^. Each of these stages is related to the function of several brain regions. The insula is a cortical region that contributes to multiple functions critical to human behavior, cognition, and emotion, and it is, as proposed by Georgiadis and Kringelbach^9^, involved in both the wanting and liking. Specifically, the anterior insula is thought to be involved in sexual desire (‘wanting’) but also, together with the posterior insula, in the ‘liking’ of sex.

Previous fMRI studies found that the anterior insula has functional connectivity (FC) with the ACC, prefrontal/frontal cortices, and temporo-parietal regions, playing a role in visceral information processing and subjective feelings^11–13^. The posterior insula has functional connectivity (FC) with the somatosensory cortices, posterior and middle cingulate, and temporo-parietal regions^14^, suggesting a more predominant role in somatosensory recognition, homeostatic processing, and the monitoring of inner bodily states. The two sub-regions are connected in a “Posterior-To-Anterior” way, with the somatosensory elaboration of a stimulus in the posterior region promoting the emotional evaluation and integration in the anterior portion of the insula^13^. This within-insula connectivity is also considered to be relevant to sexual response^10^.

General levels of activation in the insula correlated with subjective SA and genital response^3,15–17^. Conversely, tactile stimulation of the penis increased cerebral blood flow in the right posterior insula and in the adjacent secondary somatosensory cortex^18^. This role was also confirmed in studies using visual sexual stimuli^3,16,19^. According to Stoleru^6^, the posterior insula is involved in the appraisal of sexual relevant stimuli.

Recently, in a magnetoencephalographic study, Alho et al.^20^ observed the involvement of the insula in the response to stimuli depicting nudes. The activity of the insula, together with the ACC, was found between 200 and 300 ms following the stimulus presentation, a period considered relevant to the extraction of sexually relevant information from a stimulus. On the basis of these findings, a role of the insula in ‘sexual wanting’ could be hypothesized. Over the past decade, interest in the role of visual attention in the processing of sexual stimuli and sexual arousal has increased^21,22^. Earlier studies used eye-tracking, a reliable and valid measure of visual attention^23^, to investigate attentional processing in anxiety and depression^24–26^. Studies on the processing of sexual stimuli found that men spend more time than women looking at faces and genitals^27^. Moreover, a temporal pattern has been found for fixations on body parts in visual sexual stimuli^28^. Fixations tend to first involve faces and then pelvic regions. It could be assumed that detection of sexual content of stimuli would be facilitated across processing stages enabling sexual response in men. Fixations in these regions were also associated with increased physiological arousal measured by pupil dilation and subjective arousal^28^. Thus, it should be plausible to hypothesize a relationship between fixation patterns and sexual response in men.

Given the assumed role played by the insula in SA, the current study was designed to investigate functional connectivity patterns involving the anterior and posterior insular sub-regions during sexual arousal in response to visual sexual stimulation (VSS). Several studies have attempted to untangle the role played by above mentioned brain regions in male SA using different analysis approaches, investigating relevant brain regions or networks during VSS. In the current study, we hypothesize that the insula plays a role during the temporal dynamics of genital response activation^3,29^ and visual attention measured using eye-tracking methodology.

As such, this is the first exploratory study to use the combination of fMRI, eye–tracking and penile response measurement, to assess brain activation patterns in conjunction with visual attention and genital response in the study of male SA at different levels.

## Results

### Psychophysiological results

**Figure 1:**
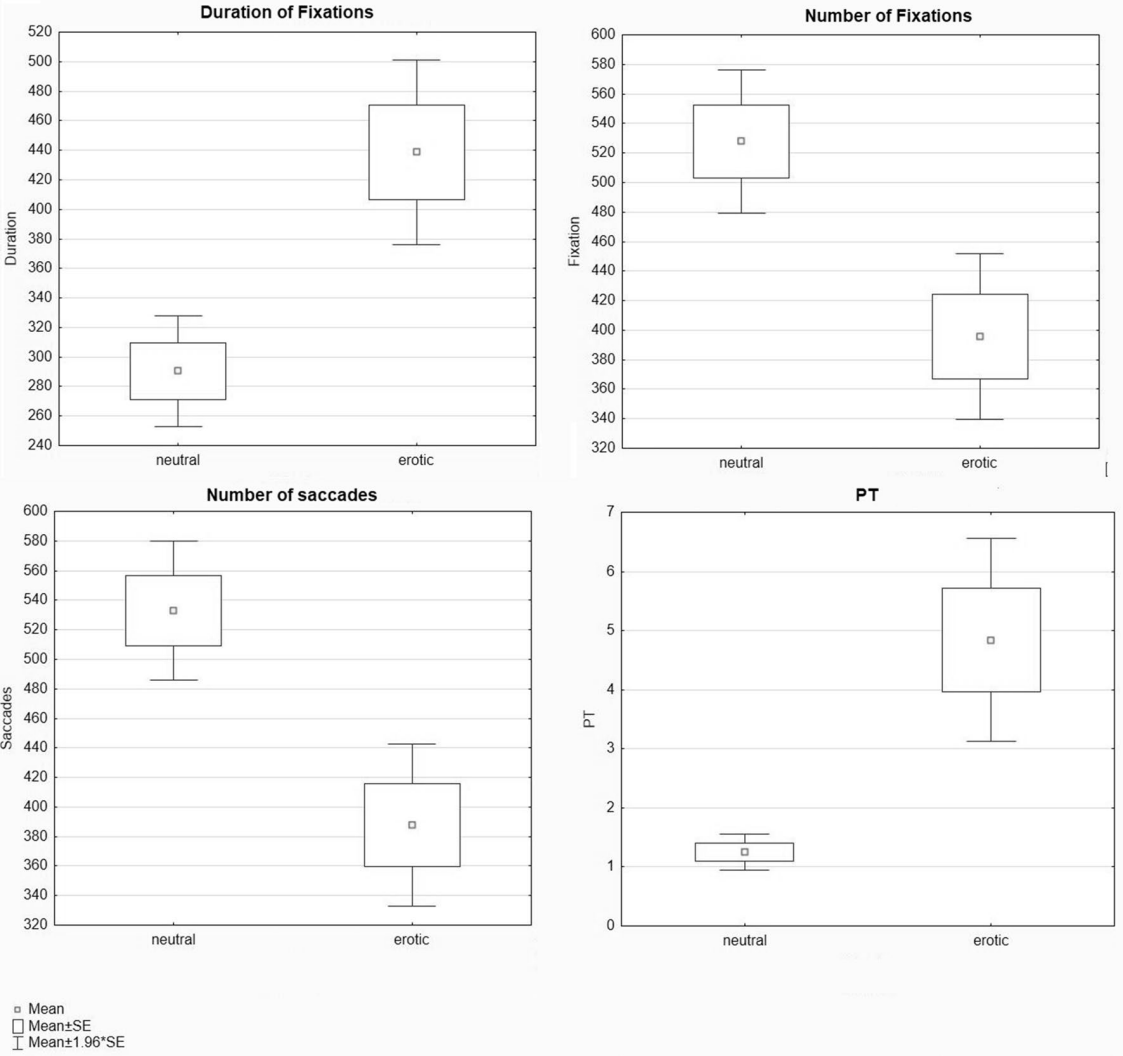
Psychophysiological results: Box and whisker plots. The figure depicts the box and whisker plots for the significant results for the psychophysiological parameters collected during the neutral and erotic clips presentations. PT, Penile Tumescence.

One way ANOVA revealed significantly higher PT (Penile Tumescence) values for the Sexual than for the Neutral conditions (F_(1,36)_ = 16.23; partial-eta- squared = 0.310; p = 0.00027—Fig. 1; Table1) .

**Table 1:** Psychophysiological data collected during the experiment.

Eye movements	Erotic		Neutral	
	Mean	Std. Err	Mean	Std. Err
Fixations (number)	395.48	27.87	527.93	24.07
Duration of fixations (ms)	438.72	31.03	290.43	18.69
Averaged pupil size (mm^2^)	2290.6	189.93	1779.08	167.77
Blinks	106.07	33.85	121.28	39.397
Duration of the blinks (ms)	136.06	19.86	135.01	16.74
Saccades (number)	387.52	27.32	532.84	23.30
Duration of saccades (ms)	72.56	10.22	60.16	6.44
Amplitude of saccades (AMPL) in degrees of visual angle	2.56	0.14	2.64	0.12
Peak velocity (degree/s)	291.10	32.80	270.11	25.19
Penile tumescence (% variation)	4.84	0.87	1.25	0.15

Also, for the eye movement data, we found that the parameters showed a significant difference (λ_(9,28)_ = 0.238; F = 9.91; partial-eta-squared = 0.761; p = 0.00000119) . The number of fixations was smaller (F_(1,36)_ = 12.25; p = 0.00125) but their duration was higher (F_(1,36)_ = 15.87; p = 0.000315) during Sexual as compared to Neutral conditions. We found a significant higher number of Saccades during the neutral condition (F_(1,36)_ = 15.51; p = 0.00036—Fig. 1; Table1). Similarly, we found a significantly smaller number of fixations for “Genitals” as compared to “Faces” (F_(1,36)_ = 7.59; partial-eta-squared = 0.174; p = 0.00914- Fig. 1). Significant correlations were found between PT and the duration of the blinks (r = 0.64 , p = 0.003, Bonf. Corr. P < 0.05), duration of saccades (r = 0.54, P < 0.015, unc.) and Peak Velocity ( r = 0.60 , p = 0.006, unc) during the erotic video.

### fMRI results: whole brain and masked GLM

**Figure 2:**
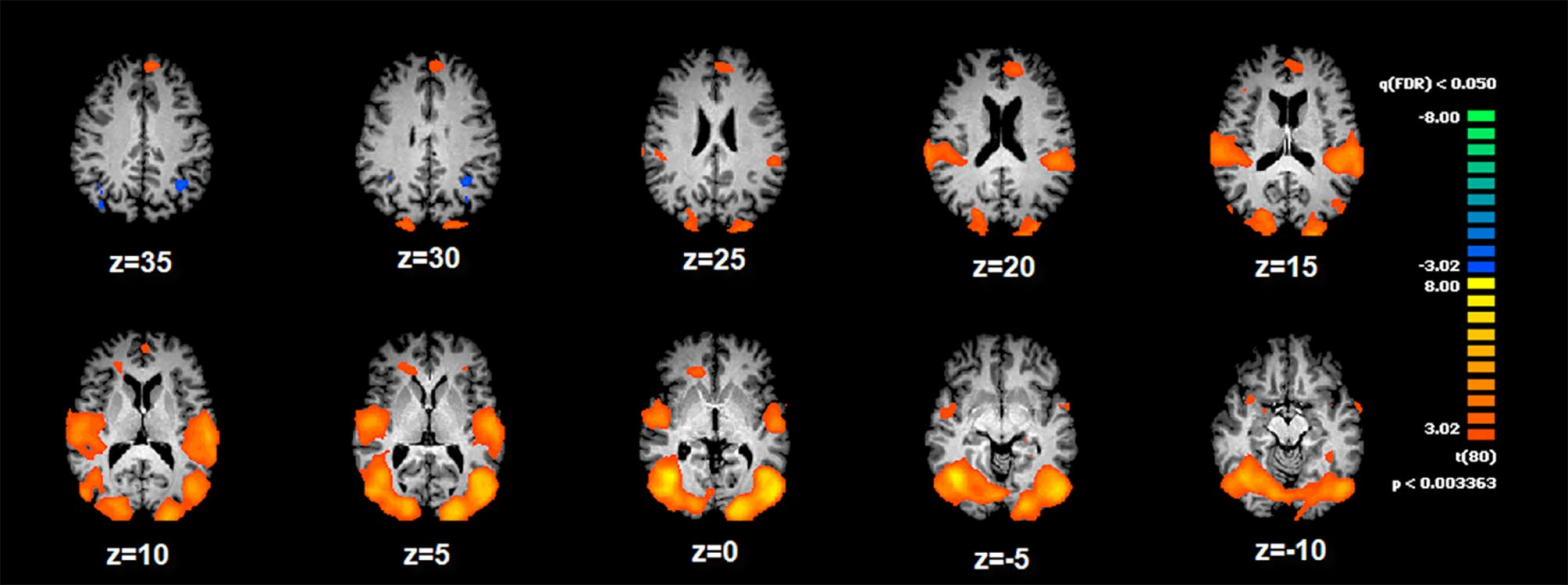
ANOVA results for Sexual > Neutral comparison. Within-group results: Cortical areas that show significant differences (in the contrast Sexual > Neutral). Red: areas more active during Sexual clips; blue: areas more active during the Neutral clips). The vertical color bar indicates t-test values. The map is thresholded at p < 0.05, FDR corrected.

The comparison between Sexual > Neutral clips revealed a significant larger activation in the bilateral posterior Insulae, supramarginal, angular gyri and anterior cingulate cortex (ACC-BA32). A larger bilateral cluster was observed in correspondence with the extra striate cortices (see Fig. 2 and Table 2). At the subcortical level, the right amygdala and temporopolar regions showed significant activation. All the results were corrected for multiple comparison (q < 0.05 FDR; p < 0.005; Fig. 2 and Table 2). The bilateral posterior insulae and left anterior insula showed a significant difference for the contrast Sexual > Neutral (p < 0.05 FDR corrected—Fig. S1). The pivotal role of the posterior insula, jointly with its adjacent was further confirmed by our whole-brain analysis.

**Table 2:** Comparison between the two epochs: Sexual > Neutral.

Cluster	BA	Side	Coordinates (mean)			Coordinates (SD)			Number of voxels	Coordinates (peak)			t values	p values
			X	Y	Z	X	Y	Z		X	Y	Z		
Striate and extrastriate cortex, Right insula, Lingual gyrus, MTG, ITG	13, 41, 22, 42, 37 18, 19,	R	9.67	− 66.5	1.13	38.29	24.71	10.99	113,209	41	− 62	− 3	8.215	0.000
SPL	7	R	35.14	− 73.9	40.08	1.86	2.28	2.58	388	35	− 74	39	− 3.478	0.00082
Temporo polar	38	R	31.14	5.02	− 13.6	2.98	2.64	2.36	617	32	7	− 15	3.594	0.000561
SPL	7	R	27.48	− 43.6	46.04	1.7	1.98	2.23	278	29	− 44	45	3.316	0.001374
Orbitofrontal	11	R	18.08	32.67	3.92	4.05	3.17	3.27	1237	14	31	0	3.820	0.000262
ACC/Frontal cortex	32 10	L	− 8.02	47.75	23.48	4.07	3.13	7.27	3085	− 13	46	21	3.781	0.000299
Somatosensory association cortex	5	L	− 10.14	− 24.4	56.98	1.88	1.49	2.03	219	− 10	− 23	57	3.199	0.00197
Primary motor cortex	39	L	− 34.58	− 56.7	33.68	2.6	2.76	2.16	575	− 34	− 56	33	− 3.794	0.00028
insula, Lingual gyrus, MTG, ITG	13,22, 41	L	− 53.58	− 26.1	9.92	8.19	14.47	6.57	21,539	− 58	− 26	9	5.828	0.00
Fusiform Gyrus	37	L	− 37.91	− 45.3	− 12	1.67	2.36	1.41	238	− 37	− 44	− 12	3.476	0.000825

Brain regions (P < 0.05 FDR corrected) are listed with the Talairach coordinates (x, left–right; y, anterior–posterior; z, dorsal–ventral) of the most significant voxel of the cluster and the corresponding t values. *BA* Brodmann’s area; *L* left; *R* right.

The FC analysis, using single FC maps for each of the two seed regions revealed different patterns for the anterior and posterior insula. FC maps for the anterior insula showed the involvement of the surrounding cortical regions belonging to the salience network and subcortical structures. Specifically, anterior insula established FC with the temporopolar regions, ACC, perigenual cingulate cortex, and bilateral inferior frontal gyrus. The FC pattern covered more dorsal regions like bilateral supplementary motor cortices (BA6), superior frontal cortex (BA8), and dlPFC. Anterior insula extended its connections to the right superior parietal lobe. Several subcortical nuclei have been found functionally connected to anterior insula, like caudate nucleus, putamen and globus pallidus (p < 0.005 Corr.).

**Figure 3:**
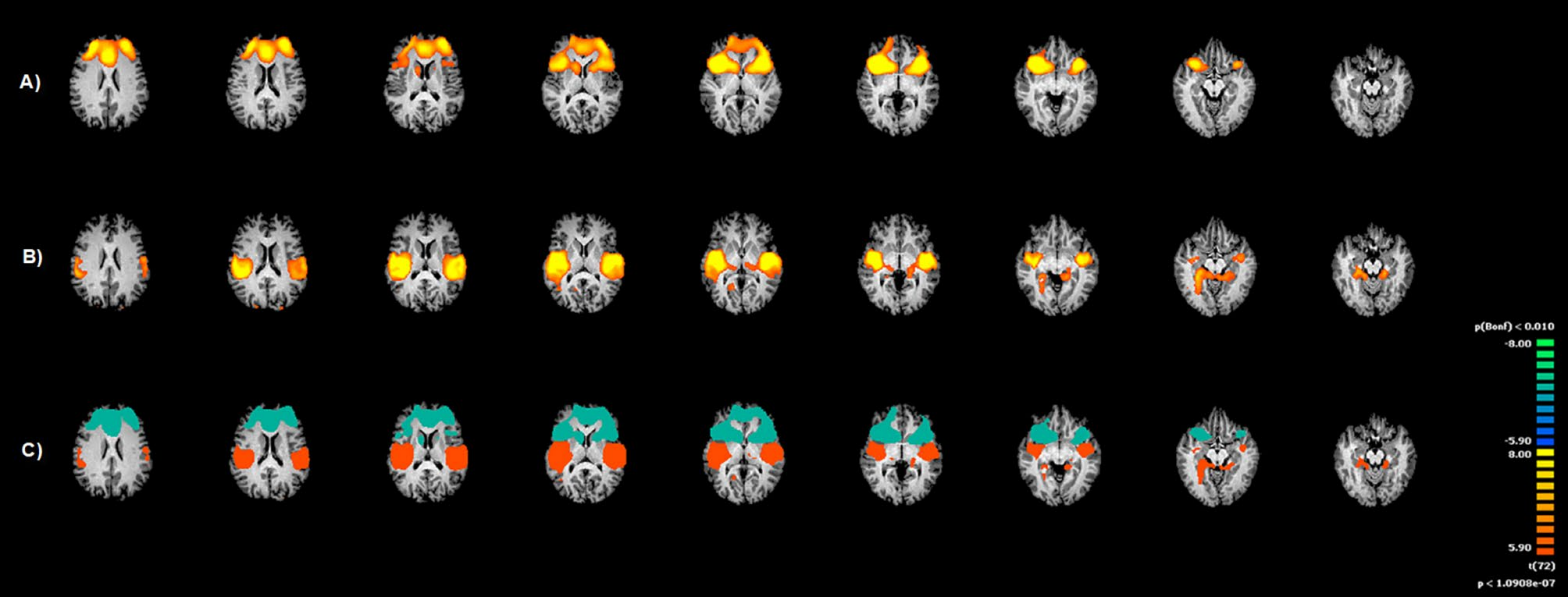
Insula functional connectivity (FC) patterns during visual sexual stimulation (VSS). Image depicts the functional connectivity patterns maps of the two sub-regions of bilateral Insula (Anterior and Posterior) as assessed with fMRI during VSS. (**A**) Functional connectivity of Anterior Insula; (**B**) Functional connectivity of Posterior Insula; (**C**) Pooled maps (light blue indicates Anterior insula Pattern, while Orange indicates Posterior insula pattern). Maps are overlaid onto a Talairach atlas and in radiological convention with a statistical significance set at p < 0.05, Bonferroni corrected.

The posterior portion showed functional connectivity with the rostro-dorsal cingulate cortex, the somatosensory regions (S2), the bilateral temporoparietal regions, angular gyri and bilateral thalamus and hypothalamus, extending to the fusiform gyru**s,** until the extrastriate cortex (Table S2-Supplementary material; Fig. 3).

After that, a series of ROI-GLMs were carried out using the eye movements data the three different phases of the genital response as above described to investigate the role played by the insula target regions in that processes.

For the comparison between areas of interest (AOIs- Genital > Faces) significant results have been found in correspondence of the target regions in the pattern of the anterior insula. Specifically, in the left anterior insula (p < 0.05 unc.), left and right IFG (p < 0.01 unc and 0.05 unc. respectively) and right SPL (p < 0.005 unc.). Similarly, for the genital response phases, we found significant activation for Onset > Baseline in the left rostral cingulate (p < 0.05 unc), left caudate nucleus (p < 0.02 unc.), and right SPL (p < 0.05 unc.). The comparison between Sustained > Baseline showed significant activation in left ACC (p < 0.005 Bonf.corr.) and left ventrolateral prefrontal cortex (p < 0.03 Bonf.corr.).

**Figure 4:**
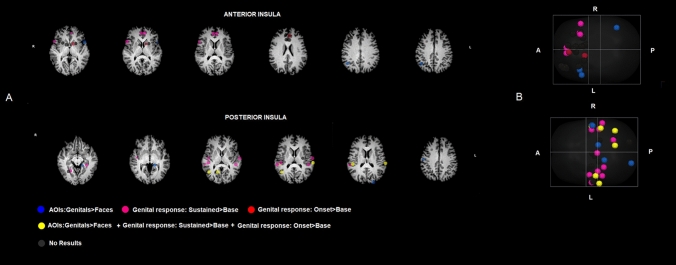
Single-ROI GLMs results. Image depicts the brain regions, spherical ROIs in which the 3 Single-ROI GLMs have been applied of the Anterior Insula Pattern and Posterior Insula Pattern. ROIs have been grouped, using different colors, on the bases of the GLMs results as described in the figure. (**A**) ROIs are overimposed on a Talairach template, which is in radiological convention (**B**) the same ROIs are overimposed on a Glass-brain in neurological convention. Brain ROIs have been created by means of TalCoord2VOI Plugin implemented in Brain voyager 2.8.

In the posterior insula target regions pattern, for the AOIs comparison (Genital > Faces) significant results have been found for the postcentral gyrus (p < 0.05 Bonf.corr.). Moreover, the right precentral gyrus (p < 0.05), posterior part of the superior temporal sulcus-gyrus/angular gyrus (p < 0.05) and the supramarginal gyrus (p < 0.05), cuneus (p < 0.05), thalamus (p < 0.05), and STG (p < 0.05); left angular ayrus (p < 0.05), posterior insula (p < 0.05) and postcentral gyrus (p < 0.05) all showed significant (but uncorrected significance level). As for genital response, we found significant results for the contrast Onset > Baseline in correspondence right angular (p < 0.05 Bonf. corr) and supramarginal gyrus (p < 0.05 Bonf. corr), cuneus (p < 0.001 Bonf. corr) and STG (p < 0.001 Bonf. Corr). For the Sustained > Baseline significant results have been observed in right angular (p < 0.001 Bonf. corr) and supramarginal gyrus (p < 0.001 Bonf. corr), cuneus (p < 0.001 Bonf. corr), STG (p < 0.001 Bonf. Corr), left supramarginal gyrus (p < 0.001 Bonf. Corr—Fig. 4). More details regarding the analyses and findings are presented in the supplementary materials (Table S1).

## Discussion

Several studies have explored the brain correlates of sexual arousal in men^3,6,17,29,30^. Previous findings highlight the role of several components, at different stages of sexual response, which all tend to rely on one or more brain regions^6^ Other studies have investigated functional connectivity and relied on the use of data derived from resting state or the presentation of visual stimuli of longer duration^30–33^. The present study investigated the functional connectivity of the insula during the presentation of visual sexual stimuli, based on analyses using both eye movement and genital response data to test the role of selected seed-ROIs, involving the anterior and posterior insulae. Our whole-brain sexual > neutral comparison, revealed that these two seed-ROIs were indeed both activated in response to visual sexual stimuli.

In the field of brain imaging and male sexual arousal, the current study is the first to combine three different measurements during presentation of sexual stimulation: fMRI, genital response, and eye movements. Moreover, the present study focuses on the role of functionally connected target regions, in terms of visual attention processing and genital response, to the two principal sub-regions of the insula. The assessment of genital response was implemented as a manipulation check and facilitated the interpretation of changes that occur at the cortical level. Despite between-subject variability in genital response induced by VSS, we observed a significant difference between the sexual and neutral conditions. This result is in line with previous results^3,16,29^. Eye movements recorded continuously during VSS showed a significant difference with fewer but longer fixations during sexual versus neutral video clips. Lykins et al.^21^ also observed a reduction in the number of fixations during erotic as compared to non-erotic stimuli. The increase in duration of fixations during the sexual clips may be related to a more focused attention to, or a stronger attentional engagement with, sexual stimuli. Thus, this increased attention with fewer fixations but of higher duration may be the result of a cognitive processing effort in seeking features in the sexual stimuli that can evoke or facilitate the maintenance of genital response.

These findings could also be related with the results from previous studies on the key role played by the attentional focus to sexual cues in the prediction of sexual response^34,35^. It has been proposed that the brain regions involved in male sexual response can be grouped in three stages^9^.The insula is considered relevant to both of the first two stages, involving wanting and liking. However, the division between a wanting-stage, involving the anterior insula, and a liking-stage, involving both the anterior and posterior insula, should, be considered artificial or, at best, tentative. Indeed, visual sexual stimulation, triggering the insula, appears to blur the temporal and spatial boundaries between these first two stages. Moreover, the wanting-stage, conceived of as being relevant to sexual desire, can rely on a very fast network. For example, in women with hypoactive sexual desire, Vardi et al.^36^ observed an altered response in the auditory p300. This could be due to the range of functions of the insula, a multimodal interface serving different cognitive and sensorial/emotional processes^12,37,38^.

Our results did show less prominent involvement of the bilateral anterior insula in SA. Visual sexual stimuli evoked a stronger and significant response in the bilateral posterior insulae. Previous studies reported the involvement of the anterior insula in several stages of male SA^6^. Anterior insula activity has been found to be coupled to activity of the ACC, both being part of the salience network^12,37,38^. ACC in the stage of wanting-sex is conceived to play the role of the network-hub, with the insula in target regions. In the present study we found the target region ACC in the network of the anterior insula. Our results are in line with previous studies on the saliency network. The anterior insula target regions are located principally in the frontal lobe and anterior portions of temporal lobes. The posterior insula pattern reflected principally the parietal, occipital, and the caudal part of temporal lobes. The FC pattern of the posterior insula is similar to that observed by Taylor et al.^14^, who used a similar anatomical location in the bilateral insulae.

Interestingly, we observed a predominant effect for the posterior insula over the anterior one. Among the target regions of the anterior insula, few showed a significant relationship with the attentional and genital processes during VSS. Instead, in the target regions of posterior insula we found three different pathways: the first is composed by a set of regions involved in the visual focused attention, onset of the erection and sustained erection; the second is only involved in processes related to the onset of erection; and the third is only related to the maintenance of or sustained erection. Moreover, we observed several regions, including in the pathway, that did not show significant associations with one or more of the three processes.

Our results confirm the involvement of the posterior insula in the recognition and monitoring of bodily sensations and feelings^39–41^. The posterior insula is therefore a region related to the somatosensory cortex in functional terms^42,43^. Among the target regions of the posterior insula, several were found to be involved in visual attention to sexual cues and genital response. Thus, we observed a pivotal multifaceted role of the posterior insula in the interplay among cognitive, somatosensory and socio-emotional functions elicited during VSS. In particular, among the posterior insula FC pattern, we observed a network of regions involved in mediating the focus of attention and in the processing of emotions, sensations, and feelings during the onset and maintenance of erection during sexual clips. Specifically, the left lateral S2 is the only one involved during attention to genitals, the onset of erection, and sustained erection. The Postcentral Sulcus/Gyrus target is involved in the eye fixation, during the onset and sustained erection. This region has been found to be involved in micturition in healthy men and possibly it could be relevant to the somatomotor representation of male genitalia^44^. We studied eye fixations effects by means of the contrast between the areas of interest located in the genitals and faces. It is conceivable that the posterior insula, which is positioned close to the precentral and postcentral gyrus, receiving visual information from the striate and extrastriate cortices, can promote an embodied representation of genital feelings during VSS^18,45–47^. That is, the posterior insula may extract sexually relevant information, coupled with the visual cortices, and helps to create an embodied representation, promoting empathy between the actor and the participant. This kind of process could have a possible explanation in the involvement of superior temporal gyrus, cuneus, and fusiform gyrus.

Despite its role in social cognition and empathy^48,49^, activation in STG has been found to be associated with clitoral stimulation^50^. According to Michels et al.^50^, the activation in superior temporal gyrus STG) was closed to temporoparietal junction. This region, considered part of the mirror system^51^, is involved in the multimodal integration of body-related information^52^.

Few regions are uniquely involved in the onset of erection; principally the occipital regions and the most part overlapped the other two functions. This could be related to the importance of visual attention as a trigger of genital arousal. The remaining target regions, showing involvement in sustained genital response, include the AG, dorsal striatum, and occipital regions. The AG is anatomically connected to other areas of the temporal lobe^7,53^. The AG can be conceived of as a cross-modal integrative region enabling us to give meaning to an event occurring in a contextualized environment (‘the here and now’). This process is reliant on prior expectations and knowledge and contributes to the planning of actions^54,55^. Such processes may have relevance to SA in that it is conceivable that the experience of having an erection provides the experience of specific feelings and affective reactions, and that those experiences themselves are based on prior experiences and emotions. During sustained erection, possible rewards could be reflected through different levels of activation in the dorsal striatum. This region, together with the putamen, is functionally connected to the posterior insula, showing a role also in the reward-response outcome association, and had been found to be related to specific personality traits such as drive-motivation^56^.

In earlier models of sexual response inspired by and based on brain imaging studies^7,8^, the posterior insula was considered part of the “emotional component,” or relegated to a marginal role with close connections to areas relevant to cognitive processing^3,6,31^. Our findings suggest a more dynamic and complex role for this specific region in male sexual response. Our multimodal approach revealed that this region plays a role in the monitoring of bodily changes, and influences other cognitive and emotional functions like visual focused attention, integration with emotional arousal, cognitive empathy, and context integration during the processing of sexual stimuli. The role of the anterior insula in sexual arousal seems to be consistent with its role within the salience network^12,37,57^. These findings shed new light on the complexity of human sexual response in the context of visual stimulation. Moreover, our findings highlight the multifaceted function played by the posterior insula in emotion processing.

### Study limitations

Despite the new insights it provides, our exploratory study has some limitations that we need to acknowledge. The most important concerns the relatively small sample size. Moreover, several studies (see Iacovella and Hasson for a review^58^) have investigated physiological processes, like cardiac and respiratory activity, or variation in blood pressure, that could affect the BOLD signal. Increasing the sample size, following the guidelines suggested by Turner et al.^59^, and controlling for possible confounding variables related to physiological noise, would allow for a more reliable and careful way to address the role played by the Insula in human sexual arousal, enhancing the results obtained in the present work.

## Methods

### Participants

Nineteen healthy men (mean age = 32.9 years, SD = 8.9**)** participated in the present study. Participants were recruited by means of advertisement in social networks and local newspapers. Each participant underwent a clinical evaluation, performed by a sexologist or clinical psychologist, to establish the absence of sexual dysfunction or psychopathological conditions. None of the participants had a history of neurological, psychiatric, or general medical diseases, and no-one was receiving any form of psychopharmacological treatment (including PDE-5 inhibitors). The study procedures were explained in detail in advance and all participants provided informed consent. The Ethics Committee of the Faculty of Medicine of the University of Coimbra approved the study protocol which was conducted in accordance with the Helsinki Declaration^60^.

### Experimental design

**Figure 5:**
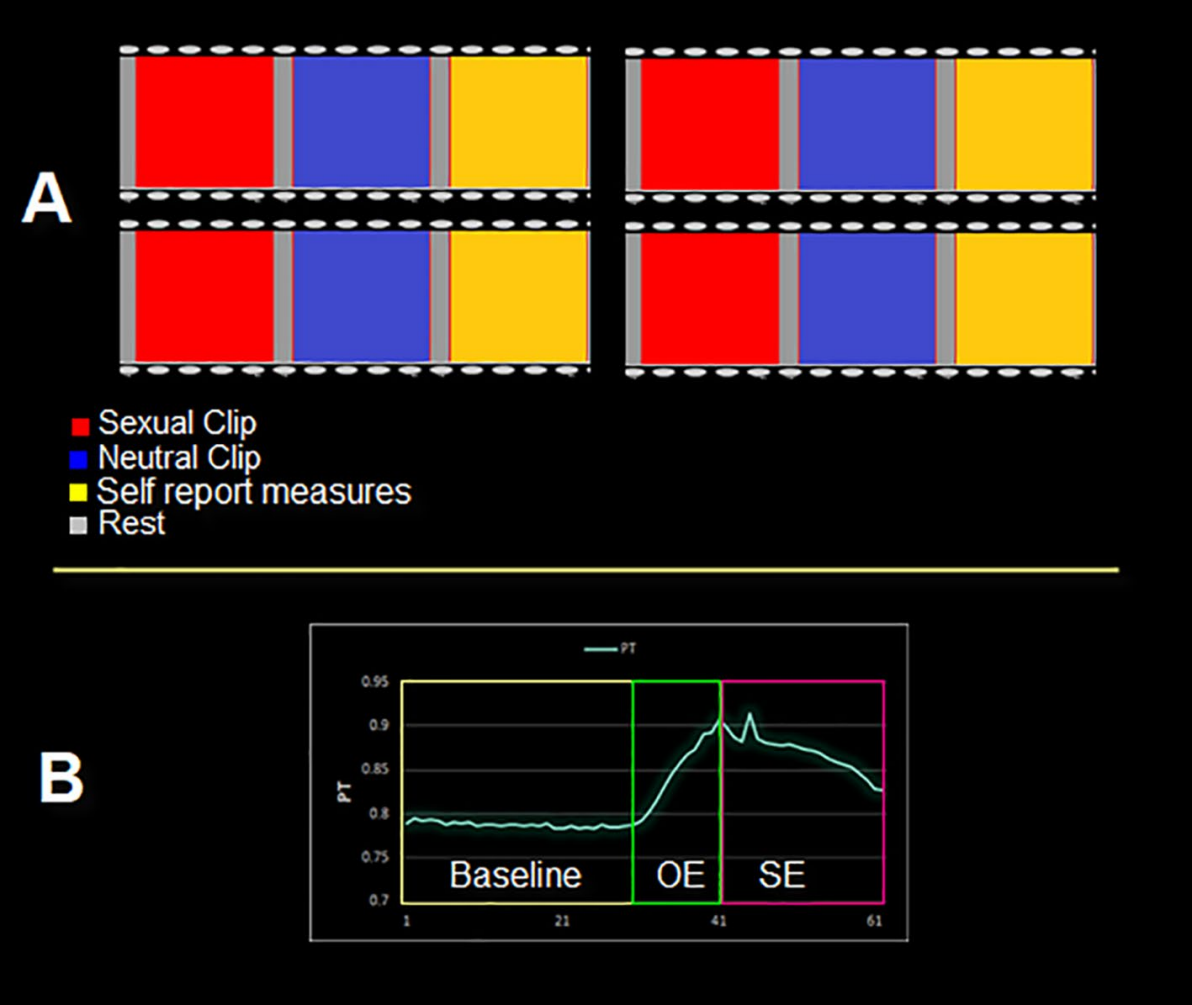
Experimental design. (**A**) Schematic representation of the experimental design consisting of a fixed order of presentation in which the sexual video clips were randomized across subjects. Each order consisted of three identical conditions, each composed of a sexual video clip (blue), a neutral video clip (red), a rest period (light gray), and self-report measures (yellow). (**B**) Example of Genital responses (PT) collected during the sexual video clip with baseline, onset of erection (OE) and sustained erection (SE).

The study involved a block design with audiovisual stimulation with 4 neutral and 4 erotic video clips (Fig. 5). Erotic clips depicted a consensual sexual interaction between a man and a woman and included petting, oral sex, and penile-vaginal intercourse. Selection of the erotic videos was based on a pilot study in which 10 erotic clips were presented, in random order, to a separate sample of healthy subjects (age 23–45 years) who rated the clips on how sexually arousing they were using seven-point Likert-like scales. The selection of neutral clips was based on previous studies from our research group^22,35^. The duration of each stimulus was 3 min, followed by an ISI, which included the instructions, of approximately 30 s. Following each sexual video presentation, participants completed a series of questions to assess subjective arousal emotions (PANAS^61^), and thoughts evoked during the visual erotic stimulation (STQ^62^ ) using Likert like scales (this part of the block design was not included in the analyses). The order of runs was determined using a random number generator. The presentation of video clips and recording of questionnaire responses was controlled by Presentation Software (Neurobehavioral Systems Inc.) on a PC placed in the console room. Stimuli were projected by means of an LCD (NordicNeuro Lab) monitor placed behind the scanner bore.

### Genital response, fMRI, and eye tracking data collection

Penile tumescence was collected during visual stimulation and fMRI data acquisition by means of a custom-built MRI-compatible pneumatic device based on a newborn-sized blood pressure cuff. The pressure cuff, which was put in place by the participant after he first put a condom on his penis, was inflated to an initial pressure of 80 mm Hg. The cuff was connected to a pressure transducer using a thin tube and the analog signal from this device was fed through an amplifier and recorded at a sampling rate of 100 Hz on a PC for offline analysis.

Eye movement data were collected using an EyeLink-1000 (SR Research Ltd.) long-range mount, binocular fMRI-compatible eye tracker, with a sampling rate of 500 Hz. Eye movement variables included fixations (number and durations), average pupil size (APS-area in mm^2^), blinks (number and durations), and saccades (number, duration, peak velocity, and visual angle covered during the saccade).

Imaging data were collected by means of a 3 T Siemens Magnetom Trio Tim MRI scanner (Siemens, Erlangen, Germany) during the tasks using a whole-body radiofrequency coil for signal excitation and a head coil for signal reception. Blood oxygen level-dependent (BOLD) fMRI data were acquired by means of T2*weighted echo-planar (EPI) sequences with the following parameters: TR of 2500 ms, TE 30 ms, FOV 230 mm, matrix size 96 × 96, voxel size 3 × 3x3mm, 360 volumes-max and 39 slices for each run, GRAPPA factor 2 left -right, flip angle 90°, no gap. A high-resolution structural volume was acquired via a 3D fast field echo T1-weighted sequence (MPRAGE-matrix = 256 × 256, FOV 256 mm, isotropic resolution of 1 mm, flip angle 7°, TR/TE = 2530/3.42 ms, Grappa factor 2- anterior posterior).

### Data analysis

#### Genital response and visual attention

Penile tumescence variation was calculated and resampled to the TR value (2.5 s). Averaged percent change normalized values were obtained. An one way ANOVA was performed to assess between-epoch differences.

Eye tracking data were analyzed using Statistica 13.3 (TIBCO Software Inc. 2017) and Matlab R2013a (Mathworks, USA). An one way MANOVA, and a series of follow-up one way ANOVAs, have been used to evaluate between-epoch differences (sexual/neutral) for all the parameters. Two areas of interest (AOIs) were manually drawn, frame-by-frame, for the 4 sexual clips, involving faces and genitals of the actors. The fixations were classified into one of three categories: Faces, Genitals, and Background. Fixation parameters (number of fixations, fixation duration min, max and mean values) were extracted for each frame of the sexual clips. An one-way ANOVAs were used to test between-AOIs differences for averaged fixation duration and number of fixations. A Pearson’s correlation was computed to assess the relationship between eye movements and PT. Missing values were replaced by mean values.

### fMRI/MRI

BOLD fMRI data were analyzed using Brain Voyager 2.4 (Brain Innovation, Maastricht, the Netherlands).

Preprocessing consisted of motion correction, linear detrending, and slice scan time correction, Fourier basis set with 2 cycles high-pass filtering. The estimated motion parameters for the different runs were inspected and compared across participants using t-tests. Preprocessed functional volumes were co-registered with the corresponding structural data set. Structural and functional volumes were then transformed into Talairach space^63,64^.

Data of the individual participants were analyzed using the single subject General Linear Model (GLM^65^), after which the estimated parameters were entered in a second level voxel-wise random effects multi study/subject analysis^66^. To assess differences between the two experimental conditions (Sexual vs Neutral), a voxel-wise repeated measure one-way ANOVA was carried out using Brain Voyager’s ANOVA tool. The baseline block was defined as the time windows with only the presence of a fixation cross (Fig. 2).

As this study focuses on the role of the insula in SA in men, a mask (ROI) was constructed in correspondence with the insulae, and the contrast between sexual and neutral stimuli was calculated following the recommendations of Ishizu & Zeki^67^ (Fig. S1—Supplementary material), following the Brodmann area13 implemented in the Brodmann.voi file in BrainVoyager.

Based on our hypotheses, supported by GLM results, we performed a seed-based functional connectivity (FC) analysis.

Each seed was selected based on the anatomy of the Insular Cortex (Tables 1, S1-supplementary material).

In particular, the insula was divided into two bilateral sub-regions, following the approach previously used by Taylor et al.^14^ and Cera et al.^68^. The two sub regions have been delineated using the the atlas from the FIND Lab at Standford University (https://findlab.stanford.edu/functional_ROIs.html), more specifically the Individual Network atlas which specifies the anterior and posterior Insula. Specifically, the anterior sub-region covers the anterior gyrus and the posterior one involves the posterior gyrus. To avoid including white matter, each seed was designed to include a 4 mm-radius sphere created by means of the TalCoord2VOI Plugin implemented in Brain voyager 2.8.

Whole-brain seed-based connectivity maps were created for all subjects. To assess the contribution of each single subject ROIs, single subject FFX-GLM, adding the mask created for the insula and the single Talairach coordinates, t and p values of the peaks are reported in Table S1 (Supplementary Material).

We then calculated correlations between seed time-courses (i.e., the insula sub-regions) and the time-courses of all brain voxels. BOLD time-courses were extracted from each ROI by obtaining an average value for each voxel of the ROI modeled for each single subject (Talairach Coordinates are described in Tables 1, S1). To examine FC patterns, after applying Fisher's r-to-z transformation to each correlation map, random-effect analyses were performed.

Based on the 2 resulting seed-based FC maps, the clusters of the target regions were selected and reported in the Table 2.

To examine the contribution of the FC target regions pattern of the anterior and posterior insula to visual attention and PT responses, we modeled the eye -fixations and PT responses using two different “single- ROI GLMs,” applied separately to each cluster of interest by means of the ROI tool of BrainVoyager.

The first GLM was created taking into account the time windows in which eye fixations landed on genital or face AOIs. For this reason, two conditions, face and genital, have been added to the model. The third condition, or baseline, has been considered as the time windows in which eye fixations landed in areas of the screen, different from the above-mentioned AOIs.

Then a multi-subject GLM was performed for the contrast Genitals > Faces using the ROIs of anterior and posterior insula > baseline (see Tables S2–S3).

A similar approach was used for the PT data. For this purpose, the PT signal was divided into three different phases, as described in Ferretti et al.^3^ and Cera et al.^29^. The first phase was the baseline genital response, as measured at the start of the sexual clip; the second one represented onset of erection (i.e., change in slope until a plateau was reached), and, finally, sustained erection corresponding with the plateau. Two specific contrasts were then calculated for each ROI: Onset > Baseline Genital State and Sustained Erection > Baseline Genital State.

## Supplementary information

Supplementary Figure.

Supplementary Information.

## Data Availability

Our data are stored in the Brain Imaging Network database (University of Coimbra, Coimbra, Portugal). These are available from the corresponding author on reasonable request.
