# Supplementary figures and images for: The role of anterior and posterior insula in male genital response and in visual attention: an exploratory multimodal fMRI study
Source: Sci Rep. 2020 Oct 28;10:18463. doi: 10.1038/s41598-020-74681-x (PMC7595210; doi:10.1038/s41598-020-74681-x)

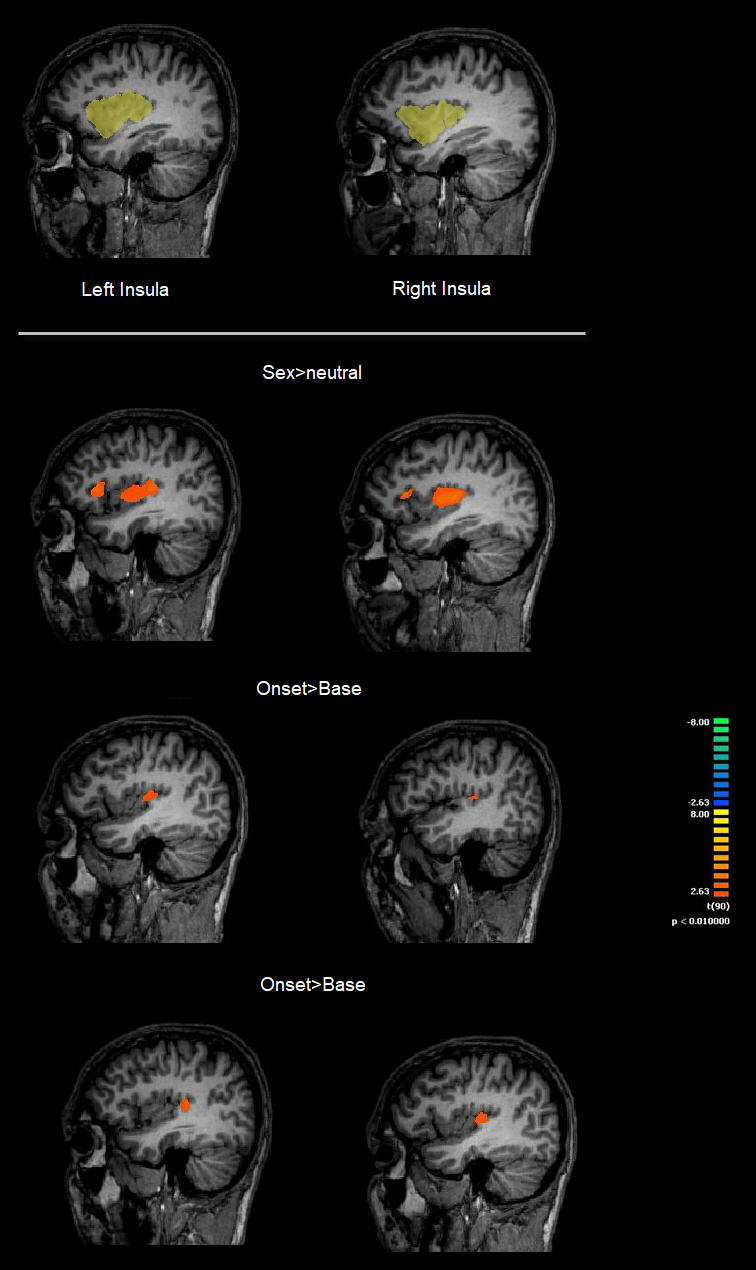

Supplement: Supplementary file 1 — Supplementary Figure. [file 41598_2020_74681_MOESM1_ESM.tif]
