# Supplementary material for: The role of anterior and posterior insula in male genital response and in visual attention: an exploratory multimodal fMRI study
Source: Sci Rep. 2020 Oct 28;10:18463. doi: 10.1038/s41598-020-74681-x (PMC7595210; doi:10.1038/s41598-020-74681-x)
Supplement: Supplementary file 2 — Supplementary Information. [file 41598_2020_74681_MOESM2_ESM.docx]

**The Role of Anterior and Posterior Insula in Male Genital Response and in Visual Attention: An Exploratory Multimodal fMRI Study**

Nicoletta Cera^1^, João Castelhano^2,3^, Cátia Oliveira^1^, Joana Carvalho^1^, Ana Luísa Quinta Gomes^1^, Maria Manuela Peixoto ^4,5^, Raquel Pereira^1^, Erick Janssen^6^, Miguel Castelo-Branco,^2,3^ and Pedro Nobre^1^

*^1^CPUP, Faculty of Psychology and Educational Sciences, University of Porto, Porto Portugal.*

*^2^CIBIT, Coimbra Institute for Biomedical Imaging and translational research, University of Coimbra, Coimbra, Portugal*

*^3^* *ICNAS, Faculty of Medicine, University of Coimbra, Coimbra, Portugal*

*^4^* *Digital Human-Environment Interaction Lab, Lusofona University, Porto, Portugal*

*^5^Centro de Investigação em Psicologia para o Desenvolvimento Positivo, Universidade Lusíada, Porto, Portugal*

*^6^Institute for Family and Sexuality Studies, Department of Neurosciences, University of Leuven, Leuven, Belgium.*

Supplementary materials

**Table S1: Talairach coordinates of the peak Insula seed ROIs for each participant with t and p values**

| PARTICIPANTS | Seed | X | Y | Z | t value | p |
| --- | --- | --- | --- | --- | --- | --- |
| 1 | Left Posterior Insula | -39 | -17 | 8 | 4.555 | 0.0000 |
|  | Left Anterior Insula | -33 | 19 | 9 | 3.135 | 0.0018 |
|  | Right Posterior Insula | 41 | -12 | 6 | 6.412 | 0.0000 |
|  | Right Anterior Insula | 38 | 16 | 3 | 3.757 | 0.0002 |
| 2 | Left Posterior Insula | -41 | -14 | 9 | 4.371 | 0.0000 |
|  | Left Anterior Insula | -37 | 18 | 4 | 0.285 | 0.7759 |
|  | Right Posterior Insula | 41 | -7 | 5 | 5.185 | 0.0000 |
|  | Right Anterior Insula | 38 | 16 | # | 1.358 | 0.1749 |
| 3 | Left Posterior Insula | -42 | -17 | 6 | 4.686 | 0.0000 |
|  | Left Anterior Insula | -34 | 19 | 0 | -2.430 | 0.0153 |
|  | Right Posterior Insula | 40 | -9 | 4 | 4.181 | 0.0000 |
|  | Right Anterior Insula | 40 | 19 | 0 | -2.122 | 0.0341 |
| 4 | Left Posterior Insula | -42 | -17 | 6 | 2.316 | 0.0214 |
|  | Left Anterior Insula | -34 | 20 | 0 | -2.930 | 0.0037 |
|  | Right Posterior Insula | 42 | -10 | 4 | 1.152 | 0.2503 |
|  | Right Anterior Insula | 40 | 16 | 6 | -2.729 | 0.0068 |
| 5 | Left Posterior Insula | -40 | -17 | 5 | 1.608 | 0.1082 |
|  | Left Anterior Insula | -34 | 18 | 6 | 2.168 | 0.0304 |
|  | Right Posterior Insula | 38 | -9 | 6 | 1.969 | 0.0492 |
|  | Right Anterior Insula | 39 | 20 | 0 | 2.099 | 0.0361 |
| 6 | Left Posterior Insula | -41 | -15 | 5 | 3.285 | 0.0011 |
|  | Left Anterior Insula | -40 | 16 | 3 | -1.017 | 0.3095 |
|  | Right Posterior Insula | 41 | -14 | 9 | 7.189 | 0.0000 |
|  | Right Anterior Insula | 37 | 13 | 6 | -2.302 | 0.0218 |
| 7 | Left Posterior Insula | -42 | -14 | 6 | 1.075 | 0.2826 |
|  | Left Anterior Insula | -37 | 16 | 2 | -1.670 | 0.0954 |
|  | Right Posterior Insula | 43 | -14 | 7 | 1.085 | 0.2785 |
|  | Right Anterior Insula | 43 | 19 | 4 | -0.994 | 0.3207 |
| 8 | Left Posterior Insula | -37 | -10 | 2 | 4.431 | 0.0000 |
|  | Left Anterior Insula | -38 | 10 | # | 3.235 | 0.0013 |
|  | Right Posterior Insula | 42 | -15 | 7 | 4.831 | 0.0000 |
|  | Right Anterior Insula | 42 | 13 | 1 | 3.967 | 0.0001 |
| 9 | Left Posterior Insula | -40 | -15 | 6 | 9.317 | 0.0000 |
|  | Left Anterior Insula | -37 | 9 | # | 2.820 | 0.0049 |
|  | Right Posterior Insula | 43 | -13 | 4 | 8.818 | 0.0000 |
|  | Right Anterior Insula | 44 | 13 | # | 9.284 | 0.0000 |
| 10 | Left Posterior Insula | -37 | -9 | 6 | 6.363 | 0.0000 |
|  | Left Anterior Insula | -37 | 10 | 1 | 5.099 | 0.0000 |
|  | Right Posterior Insula | 35 | -8 | 6 | 6.757 | 0.0000 |
|  | Right Anterior Insula | 37 | 10 | 0 | 7.336 | 0.0000 |
| 11 | Left Posterior Insula | -39 | -13 | 2 | -2.822 | 0.0050 |
|  | Left Anterior Insula | -34 | 12 | # | -2.828 | 0.0050 |
|  | Right Posterior Insula | 38 | -4 | 8 | -2.552 | 0.0111 |
|  | Right Anterior Insula | 38 | 10 | 0 | -2.978 | 0.0031 |
| 12 | Left Posterior Insula | -43 | -15 | 9 | 14.628 | 0.0000 |
|  | Left Anterior Insula | -36 | 12 | 2 | 9.478 | 0.0000 |
|  | Right Posterior Insula | 40 | -8 | 9 | 10.855 | 0.0000 |
|  | Right Anterior Insula | 39 | 13 | # | -8.495 | 0.0000 |
| 13 | Left Posterior Insula | -40 | -11 | 3 | 0.992 | 0.3216 |
|  | Left Anterior Insula | -35 | 20 | 3 | -1.922 | 0.0549 |
|  | Right Posterior Insula | 41 | -11 | 1 | 0.821 | 0.4117 |
|  | Right Anterior Insula | 42 | 15 | 0 | -1.565 | 0.1179 |
| 14 | Left Posterior Insula | -39 | -9 | 4 | 0.848 | 0.3968 |
|  | Left Anterior Insula | -39 | 19 | # | 4.379 | 0.0000 |
|  | Right Posterior Insula | 45 | -11 | 3 | 2.223 | 0.0265 |
|  | Right Anterior Insula | 35 | 22 | # | 2.150 | 0.0319 |
| 15 | Left Posterior Insula | -37 | -3 | 3 | -1.490 | 0.1370 |
|  | Left Anterior Insula | -37 | 19 | 3 | -2.080 | 0.0381 |
|  | Right Posterior Insula | 45 | -11 | 1 | 2.191 | 0.0289 |
|  | Right Anterior Insula | 38 | 20 | 2 | -3.007 | 0.0028 |
| 16 | Left Posterior Insula | -41 | -14 | 9 | 5.733 | 0.0000 |
|  | Left Anterior Insula | -37 | 15 | 5 | 5.339 | 0.0000 |
|  | Right Posterior Insula | 43 | -7 | 6 | 5.736 | 0.0000 |
|  | Right Anterior Insula | 37 | 18 | # | 5.178 | 0.0000 |
| 17 | Left Posterior Insula | -38 | -10 | 6 | -2.801 | 0.0052 |
|  | Left Anterior Insula | -36 | 13 | 1 | -2.920 | 0.0036 |
|  | Right Posterior Insula | 43 | -11 | 6 | 1.872 | 0.0616 |
|  | Right Anterior Insula | 39 | 19 | 2 | -3.131 | 0.0018 |
| 18 | Left Posterior Insula | -38 | -13 | 9 | -3.363 | 0.0008 |
|  | Left Anterior Insula | -36 | 16 | 6 | -6.538 | 0.0000 |
|  | Right Posterior Insula | 39 | 0 | 8 | -2.616 | 0.0090 |
|  | Right Anterior Insula | 35 | 15 | 6 | -6.615 | 0.0000 |
| 19 | Left Posterior Insula | -38 | -15 | 1 | -1.555 | 0.1202 |
|  | Left Anterior Insula | -39 | 12 | 7 | -1.859 | 0.0633 |
|  | Right Posterior Insula | 45 | -11 | 3 | 2.156 | 0.0314 |
|  | Right Anterior Insula | 39 | 19 | 2 | -1.680 | 0.0934 |

**Table S2. Posterior Insula Pattern: results from single –ROI GLMs**

|  |  | Thalairach coordinates | | | EYE(Gentals>Face) | | | PT: Onset>baseline | | | PT: Sustained>baseline | | |
| --- | --- | --- | --- | --- | --- | --- | --- | --- | --- | --- | --- | --- | --- |
| Hemisphere | Region | X | Y | Z | t | p | p Bonf. Corr. | t | p | p BonfCorr. | t | p | p BonfCorr. |
| R | STG | 51 | -11 | 3 | 2.67 | **0.01** | 0.23 | -4.13 | 0.00 | 0.00 | 4.11 | 0.00 | 0.00 |
| L | Left posterior cingulate gyrus | -2 | -22 | 45 | 2.42 | 0.02 | 0.46 | 0.75 | 0.45 | 13.61 | 3.11 | 0.00 | 0.06 |
| L | rostro-dorsal anterior cingulate cortex | -3 | -12 | 41 | 1.38 | 0.17 | 5.03 | 0.83 | 0.41 | 12.15 | 1.60 | 0.11 | 3.27 |
| L | Thalamus | -8 | -17 | 4 | 1.05 | 0.29 | 8.78 | -0.69 | 0.49 | 14.78 | -0.13 | 0.90 | 26.95 |
| L | Lateral Thalamus | -19 | -25 | 1 | 1.15 | 0.25 | 7.46 | -0.49 | 0.63 | 18.82 | 1.78 | 0.07 | 2.25 |
| R | Thalamus ventro posterior | 14 | -24 | 3 | 2.13 | 0.03 | 0.99 | -1.31 | 0.19 | 5.76 | 1.10 | 0.27 | 8.09 |
| R | parahippocampal gyrus | 23 | -27 | -12 | 1.47 | 0.14 | 4.23 | 1.39 | 0.16 | 4.94 | 1.11 | 0.27 | 8.07 |
| L | MTG | -45 | -7 | -10 | 0.84 | 0.40 | 12.11 | 1.55 | 0.12 | 3.67 | 2.45 | 0.01 | 0.43 |
| L |  | -38 | -23 | 15 | 2.40 | 0.02 | 0.49 | 1.55 | 0.12 | 3.64 | 7.29 | 0.00 | 0.00 |
| L | postcentral gyrus S2 | -47 | -19 | 42 | 3.37 | 0.00 | 0.02 | 2.95 | 0.00 | 0.10 | 2.46 | 0.01 | 0.42 |
| L | parahippocampal gyrus | -16 | -36 | -4 | 1.06 | 0.29 | 8.74 | 2.19 | 0.03 | 0.87 | 2.75 | 0.01 | 0.18 |
| L | Superior occipital gyrus | -22 | -89 | 19 | 0.16 | 0.87 | 26.11 | -2.22 | 0.03 | 0.79 | 1.09 | 0.28 | 8.31 |
| L | fusiform gyrus | -30 | -35 | -11 | 1.87 | 0.06 | 1.84 | 3.30 | 0.00 | 0.03 | 3.18 | 0.00 | 0.04 |
| L | Middle postcentral gyrus | -58 | -14 | 32 | 0.76 | 0.45 | 13.41 | -0.45 | 0.65 | 19.50 | -2.68 | 0.01 | 0.22 |
| L | STG | -62 | -26 | 15 | 2.06 | 0.04 | 1.19 | -2.88 | 0.00 | 0.12 | 4.25 | 0.00 | 0.00 |
| L | Heschl's gyrus | -60 | -11 | 11 | 1.17 | 0.24 | 7.28 | -1.72 | 0.08 | 2.54 | 3.89 | 0.00 | 0.00 |
| L | planum temporal | -47 | -32 | 10 | 2.79 | 0.01 | 0.16 | 0.34 | 0.74 | 22.08 | 10.88 | 0.00 | 0.00 |
| R |  | 24 | -54 | -7 | 1.57 | 0.12 | 3.49 | 1.96 | 0.05 | 1.51 | 3.43 | 0.00 | 0.02 |
| R | fusiform gyrus/lateral Occipital | 42 | -57 | 10 | 2.00 | 0.05 | 1.36 | -3.40 | 0.00 | 0.02 | 4.34 | 0.00 | 0.00 |
| R | Heschl's gyrus/middle insula | 44 | -11 | 4 | 1.32 | 0.19 | 5.64 | 0.26 | 0.79 | 23.84 | 4.51 | 0.00 | 0.00 |
| R | STG | 47 | -29 | 17 | 2.47 | 0.01 | 0.41 | -3.38 | 0.00 | 0.02 | 6.81 | 0.00 | 0.00 |
| R | fusiform gyrus/lateral Occipital | 30 | -56 | -10 | 1.33 | 0.18 | 5.52 | -0.10 | 0.92 | 27.60 | 2.63 | 0.01 | 0.25 |
| R | S2 | 53 | -12 | 28 | 2.18 | 0.03 | 0.87 | -0.22 | 0.83 | 24.79 | -0.13 | 0.90 | 26.95 |
| R |  | 45 | -26 | 10 | 1.84 | 0.07 | 1.98 | -11.58 | 0.00 | 0.00 | 8.28 | 0.00 | 0.00 |
| R | Putamen/dorsal striatum | -28 | -11 | 10 | 1.38 | 0.17 | 5.02 | 0.18 | 0.86 | 25.75 | 2.23 | 0.03 | 0.78 |
| R | Frontal eye fields | 52 | -13 | 31 | 2.41 | 0.02 | 0.48 | -0.02 | 0.98 | 29.33 | -0.93 | 0.35 | 10.64 |
| R | Occipital cortex | 20 | -85 | 23 | 0.75 | 0.45 | 13.60 | -2.96 | 0.00 | 0.09 | 2.50 | 0.01 | 0.37 |
| R | Cuneus | 13 | -57 | 6 | 2.06 | 0.04 | 1.19 | 5.17 | 0.00 | 0.00 | 3.82 | 0.00 | 0.00 |
| R | Posterior part/superior temporal sulcus-gyrus | 57 | -32 | 13 | 1.96 | 0.05 | 1.51 | -1.96 | 0.05 | 1.50 | 9.65 | 0.00 | 0.00 |
| L | STS /STG | -54 | -11 | -2 | 0.84 | 0.40 | 12.11 | 1.55 | 0.12 | 3.67 | 2.45 | 0.01 | 0.43 |

**Table S3. Anterior Insula Pattern: results from single –ROI GLMs**

|  |  | Thalairach coordinates | | | EYE(Gentals>Face) | | | PT: Onset>baseline | | | PT: Sustained>baseline | | |
| --- | --- | --- | --- | --- | --- | --- | --- | --- | --- | --- | --- | --- | --- |
| Hemisphere | Region | X | Y | Z | t | P | p Bonf Corr. | t | P | p Bonf Corr. | t | P |  |
| L |  | -40 | 18 | -1 | 2.2 | 0.02 | 0.75 | -1.12 | 0.26 | 7.02 | 0.59 | 0.55 | 14.93 |
| L | IFG/Broca's area | -50 | 14 | 4 | 2.7 | 0.0 | 0.18 | -1.01 | 0.31 | 8.42 | 2.10 | 0.03 | 0.95 |
| R | Left Middle Frontal Gyrus | -37 | 44 | 7 | 0.6 | 0.54 | 14.73 | -1.66 | 0.09 | 2.58 | -0.71 | 0.47 | 12.79 |
| L | Frontal cortex | -36 | 50 | 15 | 1.5 | 0.12 | 3.25 | -0.95 | 0.33 | 9.15 | -0.23 | 0.81 | 22.01 |
| L | Putamen | -21 | 9 | 7 | 0.32 | 0.74 | 20.21 | 1.04 | 0.29 | 8.05 | 1.29 | 0.19 | 5.29 |
| L | Caudate Nucleus | -12 | 8 | 5 | -1.75 | 0.07 | 2.15 | -2.35 | 0.01 | 0.49 | -0.77 | 0.44 | 11.89 |
| R | fronto-medial gyrus | 6 | 14 | 55 | 0.87 | 0.38 | 10.27 | 1.13 | 0.25 | 6.92 | 0.89 | 0.37 | 9.99 |
|  | Superior Frontal Gyrus | 1 | 30 | 42 | 1.38 | 0.16 | 4.51 | 0.23 | 0.81 | 22.05 | 1.25 | 0.20 | 5.62 |
| R | Mesial frontal gyrus | 30 | 17 | 41 | 1.18 | 0.23 | 6.39 | 1.64 | 0.09 | 2.69 | 2.55 | 0.01 | 0.28 |
| L | Prefrontal Cortex | -33 | 20 | 39 | 1.14 | 0.25 | 6.83 | 0.92 | 0.35 | 9.62 | 0.47 | 0.63 | 17.12 |
| L | dorsal anterolateral prefrontal cortex | -32 | 54 | 17 | 0.55 | 0.57 | 15.65 | -0.08 | 0.93 | 25.19 | -0.32 | 0.74 | 20.06 |
| L | frontal polar cortex | -34 | 46 | 11 | 1.27 | 0.20 | 5.50 | -1.45 | 0.14 | 3.93 | -0.15 | 0.87 | 23.72 |
| R | SFG/MFG | 31 | 31 | 31 | 0.60 | 0.54 | 14.77 | 0.78 | 0.43 | 11.68 | 1.4 | 0.16 | 4.36 |
| R | ACC | 4 | 44 | 12 | 1.86 | 0.06 | 1.67 | 0.23 | 0.81 | 22.01 | 4.38 | 0.00 | 0.00 |
| L | ACC | -7 | 44 | 10 | 1.11 | 0.26 | 7.12 | -0.86 | 0.38 | 10.43 | 3.09 | 0.00 | 0.05 |
| L | cingulate gyrus | -5 | 25 | 24 | 1 | 0.31 | 8.44 | -1.85 | 0.06 | 1.73 | 0.90 | 0.36 | 9.92 |
| R | cingulate gyrus | 6 | 26 | 24 | 0.34 | 0.73 | 19.80 | -0.89 | 0.37 | 10.04 | 1.24 | 0.21 | 5.77 |
| L | Rostral cingulate | -5 | 39 | 22 | 1.31 | 0.17 | 4.59 | -2.18 | 0.02 | 0.78 | 1.52 | 0.12 | 3.41 |
| R | anterior cingulate dorsal | 8 | 41 | 21 | 1.37 | 0.17 | 4.59 | 1.52 | 0.12 | 3.41 | 1.52 | 0.12 | 3.41 |
| R | Ventral striatum/caudate | 7 | 12 | -1 | -0.31 | 0.75 | 20.36 | -1.36 | 0.17 | 4.64 | -1.36 | 0.17 | 4.64 |
| R | SPL | 43 | -55 | 33 | 3.03 | 0.00 | 0.06 | 2.29 | 0.02 | 0.58 | 1.10 | 0.27 | 7.29 |
| R | putamen (medial)/globus pallidus | 23 | 11 | 3 | 0.73 | 0.46 | 12.46 | 0.98 | 0.32 | 8.70 | 1.07 | 0.28 | 7.63 |
| R |  | 34 | 16 | 2 | 1.15 | 0.24 | 6.70 | 0.22 | 0.82 | 22.19 | 1.87 | 0.06 | 1.63 |
| R | IFG | 51 | 16 | 9 | 2.08 | 0.03 | 1.00 | 0.34 | 0.72 | 19.69 | 2.36 | 0.01 | 0.49 |
| R | Anterior temporal and orbitofrontal cortex | -37 | 16 | -8 | 0.62 | 0.53 | 14.46 | -0.58 | 0.56 | 15.16 | 0.38 | 0.69 | 18.85 |
|  | ventrolateral prefrontal cortex | 33 | 18 | -10 | 0.82 | 0.40 | 11.04 | 0.86 | 0.38 | 10.51 | 3.34 | 0.00 | 0.02 |
| R | ventral striatum/Globus pallidus | 10 | 5 | 5 | -0.33 | 0.73 | 19.88 | -1.27 | 0.20 | 5.50 | -1.61 | 0.10 | 2.87 |

**FIGURE S1: voxel wise one way ANOVA performed on the insula**
